# Urinary exosome microRNA signatures as a noninvasive prognostic biomarker for prostate cancer

**DOI:** 10.1038/s41525-021-00212-w

**Published:** 2021-06-11

**Authors:** Sun Shin, Yong Hyun Park, Seung-Hyun Jung, Sun-Hee Jang, Mee Young Kim, Ji Youl Lee, Yeun–Jun Chung

**Affiliations:** 1 Departments of Microbiology, College of Medicine, The Catholic University of Korea, Seoul, Republic of Korea; 2 Precision Medicine Research Center, College of Medicine, The Catholic University of Korea, Seoul, Republic of Korea; 3 Integrated Research Center for Genome Polymorphism, College of Medicine, The Catholic University of Korea, Seoul, Republic of Korea; 4 Departments of Urology, College of Medicine, The Catholic University of Korea, Seoul, Republic of Korea; 5 Catholic Cancer Research Institute, College of Medicine, The Catholic University of Korea, Seoul, Republic of Korea; 6 Departments of Biochemistry, College of Medicine, The Catholic University of Korea, Seoul, Republic of Korea

**Keywords:** Prognostic markers, Prostate cancer

## Abstract

Predicting the risk of metastasis before starting prostate cancer (PCa) treatment can minimize the overtreatment of indolent cases and help choosing appropriate treatment. The levels of circulating microRNAs (miRNAs) from body fluids can be used as noninvasive prognostic biomarkers. In this study, urinary exosomal miRNA expression profiles of 149 PCas were determined and the miRNAs associated with metastasis were identified: miR-21, miR-16, miR-142-3p, miR-451, and miR-636. When evaluating clinical factors together, miR-21, miR-451, miR-636, and preoperative prostate-specific antigen (PSA) level remained significant in the multivariate analysis. Based on them, we developed a “Prostate Cancer Metastasis Risk Scoring (PCa-MRS)” model. The PCa-MRS showed superior stratification power (AUC = 0.925) to preoperative PSA or clinical Gleason score. Patients with high scores showed significantly poorer biochemical recurrence-free survival than those with low scores (*P* = 6.53 × 10^−10^). Our results showed the potential of urinary exosomal miRNAs as noninvasive markers for predicting metastasis and prognosis in PCa patients.

## Introduction

Prostate cancer (PCa) is the second most common malignancy in men worldwide. PCa shows diverse clinical features ranging from a clinically insignificant cancer to an aggressive cancer with metastasis, castration resistance, and poor prognosis. Prostate-specific antigen (PSA) is the most well-known biomarker for PCa. However, the use of PSA remains controversial because of its low specificity and unclear relationship with both the tumor stage and grade^1^. The Gleason score (GS) is a significant prognostic factor in PCa, and higher GS has been consistently reported to be associated with poor prognosis including metastasis and PCa-specific mortality^2^. However, it is invasive as it needs tissue specimen from biopsy or prostatectomy. Although a high proportion of PCa is clinically indolent, the incidence of high-grade and metastatic PCa is increasing^2^. Thus, prediction of poor prognosis will be valuable in the early stage of treatment.

Cancer cells release exosomes into biological fluids such as urine, which are protected from degradation by the exosomal lipid bilayer. Because exosomes contain tumor-driven molecules and given the convenient anatomical location of the prostate, urinary exosomes have been considered to be ideal substrates for developing noninvasive biomarkers^3^. Exosomes contain microRNAs (miRNAs), which are small noncoding RNAs involved in the regulation of gene expression. Exosomal miRNAs have been suggested to play important roles in carcinogenesis^4^, and some miRNA signatures with diagnostic potential have been identified in several types of cancers including PCa^5,6^. Therefore, urinary exosomal miRNAs have great potential as noninvasive biomarkers for identifying patients at increased risk of developing aggressive PCa.

This study aimed to assess the utility of urinary exosomal miRNAs as noninvasive prognostic biomarkers for PCa by observing differentially expressed miRNAs between localized and metastatic PCas and exploring their biological implications. By combining significant urinary miRNA markers and clinical factors, we developed a scoring model for predicting the risk of PCa metastasis.

## Results

### Urinary exosomal miRNAs related to PCa metastasis

Urine samples from a total of 149 PCa patients were evaluated. Clinical data including preoperative PSA, PSA density, and clinical GS (cGS) are presented in Supplementary Table 1. To discover urinary exosomal miRNAs associated with PCa metastasis, we performed a two-phase approach: (I) discovery and independent validation of candidate miRNA, and (II) model construction with internal cross-validation and external validation of the model. The overall study design is illustrated in Supplementary Fig. 1. In the discovery phase, we analyzed miRNA expression profiles of 42 patients (19 localized and 23 metastatic) using the Taqman low-density miRNA array (TLDA) containing 381 miRNAs. Of the 16 differentially expressed miRNAs (Supplementary Table 2), six showed significant difference (Table 1). Their relative expression is shown in Supplementary Fig. 2. Five (miR-451, miR-142-3p, miR-16, miR-21, and miR-140-3p) were upregulated, and one (miR-636) was downregulated in the metastatic group. As a technical validation of the six candidate miRNAs, target miRNA-specific quantitative reverse transcription–polymerase chain reaction (qRT-PCR) was performed with the same samples and all of them showed consistent expression patterns and the differences were statistically significant with one exception (miR-636) (Table 1).

**Table 1:** Urinary exosomal miRNAs differentially expressed between metastatic and localized PCa.

	Discovery set				Validation set (qRT-PCR)		Combined: model construction set (qRT-PCR)	
	Discovery (TLDA)		Technical validation (qRT-PCR)					
miRNA	Fold change	*p*-value	Fold change	*p*-value	Fold change	*p*-value	Fold change	*p*-value
miR-636	0.33	**0.039**	0.82	0.582	0.44	**0.013**	0.53	**0.004**
miR-140-3p	2.17	**0.017**	2.62	**0.005**	1.76	0.137	1.19	0.424
miR-21	3.15	**0.005**	2.95	**0.003**	2.56	**0.044**	2.20	**0.006**
miR-16	2.92	**0.050**	3.31	**0.006**	1.98	0.190	2.12	**0.010**
miR-142-3p	4.33	**0.002**	11.7	**7.92** **×** **10**^−**6**^	4.83	0.206	3.20	**0.021**
miR-451	3.88	**0.049**	8.42	**0.003**	3.84	**0.008**	6.31	**2.12** **×** **10**^−**6**^

Statistically significant differences (*P* < 0.05) are in bold.

Discovery set: 42 PCa patients (19 localized and 23 metastatic).

Validation set: 70 PCa patients (56 localized and 14 metastatic).

Model construction set (Combined/qRT-PCR): 112 PCa patients (75 localized and 37 metastatic).

*TLDA* TaqMan low-density miRNA array, *qRT-PCR* quantitative reverse transcription–polymerase chain reaction.

### Validation of the candidate miRNAs

For independent validation of the candidate miRNAs, we performed target miRNA-specific qRT-PCR with an independent set of 70 PCa patients (validation set; 56 localized and 14 metastatic). Five miRNAs showed the consistent trends of expression with those in the discovery set, and three of them (miR-636, miR-21, and miR-451) remained significant in the validation set (Table 1). When we combined the data from the discovery and validation sets (112 patients; 75 localized and 37 metastatic PCas), five miRNAs (miR-636, miR-21, miR-16, miR-142-3p, and miR-451) were significant (Table 1).

### Developing a risk-scoring model for PCa metastasis

**Fig. 1:**
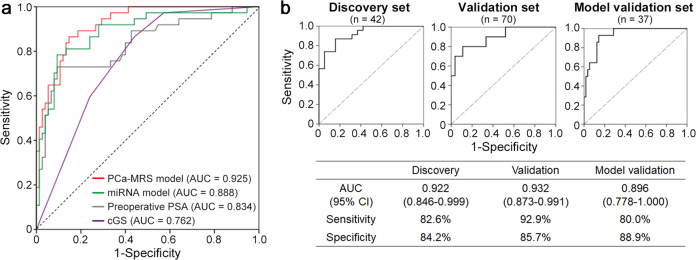
Performance of the risk prediction model. **a** Receiver operating characteristic (ROC) curves for the risk prediction model (PCa-MRS) comprising three miRNAs (miR-636, miR-21, and miR-451) with preoperative PSA level (red). The model showed superior stratification power (AUC = 0.925) to other models composed of either miRNAs (green), preoperative PSA level (gray), or clinical Gleason score (purple) only. **b** ROC curves for PCa-MRS of each sample set. Sensitivity and specificity were calculated based on a cutoff value of −0.82 in the PCa-MRS model from Youden’s index. PSA prostate-specific antigen, AUC area under the curve, cGS clinical Gleason score.

To develop a risk prediction model for PCa metastasis, we assessed the various clinical and molecular factors for their associations with metastasis in the combined data sets (Table 2). In the univariate analysis, the decreased expression of miR-636 and increased expressions of miR-21, miR-16, miR-142-3p, and miR-451 were significantly associated with metastasis (odds ratio (OR): 0.65 [95% confidence interval (CI): 0.48–0.88], 1.31 [1.07–1.61], 1.34 [1.07–1.66], 1.12 [1.01–1.24], and 1.42 [1.20–1.68], respectively). Of the clinical variables (age, body mass index (BMI), preoperative PSA, prostate volume, PSA density, and cGS), the preoperative PSA (OR: 1.04 [95% CI: 1.02–1.06]), PSA density (OR: 3.59 [95% CI: 1.58–8.12]), and higher cGS were significantly associated with metastasis (Table 2). We performed the multivariate logistic regression with backward selection using the factors that were significant in the univariate analysis. We did not include the PSA density and cGS for the regression analysis because we intended to avoid the multi-collinearity effect with preoperative PSA for developing a minimally invasive model. Among them, miR-636, miR-21, miR-451, and preoperative PSA (adjusted OR: 0.37 [95% CI: 0.23–0.61], 1.65 [1.17–2.31], 1.48 [1.15–1.91], and 1.03 [1.01–1.05], respectively) remained significant (Table 2). For model construction, we performed 100 iterations of Lasso logistic regression with five-fold cross-validation, and the median area under curve (AUC) of models was 0.917 (range 0.909–0.925) (Supplementary Fig. 3). Based on these results, we developed a PCa metastasis risk-scoring (PCa-MRS) model with the highest AUC among them, consisting of three miRNAs (miR-636, miR-21, and miR-451) and preoperative PSA as follows: Logit (P) = −8.72 + (0.97 × ΔCt of miR-636) + (−0.49 × ΔCt of miR-21) + (−0.38 × ΔCt of miR-451) + (0.030 × preoperative PSA). In the receiver operating characteristic (ROC) analyses, the PCa-MRS model showed higher discriminatory power (AUC = 0.925, 95% CI: 0.878–0.971) than each miRNA marker (AUC = 0.658 for miR-21, AUC = 0.768 for miR-451, and AUC = 0.666 for miR-636). The PCa-MRS model was superior to the models composed of the all five candidate miRNAs (AUC = 0.888, 95% CI: 0.818–0.957) or preoperative PSA only (AUC = 0.834, 95% CI: 0.749–0.919) (Fig. 1a). The PCa-MRS model also showed superior discriminatory power to the cGS (AUC = 0.762, 95% CI: 0.674–0.849) (Fig. 1a). Regarding specificity and accuracy, the PCa-MRS model also showed better performance than other models. For example, when the sensitivity threshold was set to 86.5%, the specificity and accuracy of the PCa-MRS model were 85.3% and 85.7%, whereas it was only 76.0% and 79.5% in the miRNA-only model, 57.3% and 67.0% in the preoperative PSA model, and 56.0% and 66.1% in the cGS model, respectively. Estimates of sensitivity, specificity, and accuracy for each model at specified thresholds are summarized in Supplementary Table 3.

**Table 2:** Logistic regression results for clinical variables and five miRNAs with PCa metastasis.

	Univariate		Multivariate^a^	
Variable	OR (95% CI)	*P*-value	aOR (95% CI)	*P*-value
Age	1.06 (1.00–1.12)	0.067		
BMI	1.09 (0.95–1.26)	0.230		
Preoperative PSA	1.04 (1.02–1.06)	4.63 × 10^−4^	1.03 (1.01–1.05)	0.004
Prostate volume	1.02 (0.99–1.05)	0.118		
PSA density^b^	3.59 (1.58–8.12)	0.002		
Clinical Gleason score^b^		0.004		
7 (3 + 4) vs ≤6	14.7 (1.45–148.0)	0.023		
7 (4 + 3) vs ≤6	22.0 (2.58–187.8)	0.005		
≥8 vs ≤6	40.3 (5.02–324.4)	5.09 × 10^−4^		
miR-636	0.65 (0.48–0.88)	0.005	0.37 (0.23–0.61)	9.92 × 10^−5^
miR-21	1.31 (1.07–1.61)	0.009	1.65 (1.17–2.31)	0.004
miR-16	1.34 (1.07–1.66)	0.010		
miR-142-3p	1.12 (1.01–1.24)	0.035		
miR-451	1.42 (1.20–1.68)	5.13 × 10^−5^	1.48 (1.15–1.90)	0.002

112 PCa cases (75 localized and 37 metastatic) were used for analysis.

*OR* odds ratio, *CI* confidence interval, *aOR* adjusted odds ratio.

^a^Multivariate analysis was performed by logistic regression analysis with backward selection for preoperative PSA, miR-636, miR-21, miR-16, miR-142-3p, and miR-451.

^b^PSA density and clinical Gleason score were excluded for multivariate analysis.

We further tested the performance of the PCa-MRS model with additional 37 PCa patients (27 localized and 10 metastatic PCa) as an external validation of the model. The AUC of the PCa-MRS model in the model validation set was 0.896 (95% CI: 0.778–1.000), which was compatible with the AUC of combined set (0.925, *n* = 112) described above, and those of discovery set (0.922, *n* = 42), and validation set (0.932, *n* = 70) (Fig. 1b).

### PCa-MRS score correlated with biochemical recurrence (BCR)-free survival

**Fig. 2:**
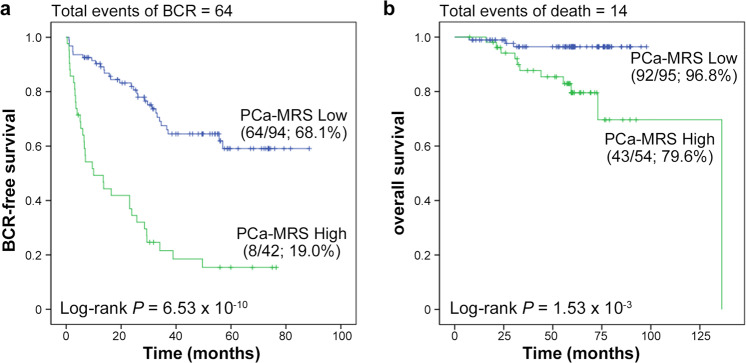
Kaplan–Meier curves for BCR-free and overall survivals by risk score. Patients with high PCa-MRS scores (green) showed significantly poorer BCR-free survival (**a**) and overall survival (**b**) than those with low scores (blue). The survival rates (survival/number of cases) are shown in parentheses. Among the 149 combined data from discovery, validation, and external model validation sets, we examined 136 patients who received radical prostatectomy (RP) for BCR-free survival and all 149 patients for overall survival. BCR biochemical recurrence.

We then examined whether the PCa-MRS score could be an independent predictor of BCR-free survival using combined data from discovery, validation, and external model validation sets (*n* = 149). Among the 149 PCa patients, we examined 136 PCa patients who received radical prostatectomy (RP) for recurrence. The median duration of follow-up was 28.5 months (range, 0.37–88.5 months). BCR-free survival was measured from the date of RP to relapse, which was defined as postoperative PSA value ≥0.2 ng/mL (a lower detection limit of 0.004 ng/mL) and successive PSA ≥ 0.2 ng/mL after surgery, or to the date of last follow-up. According to this criterion, 64 patients developed BCR. Cox regression analyses were performed using the following covariates; age, BMI, prostate volume, preoperative PSA, PSA density, cGS, and PCa-MRS score. In univariate analysis, the PCa-MRS score was found to be the most significant prognostic factor for BCR (HR: 4.23 [95% CI: 2.58–6.96], *P* = 1.25 × 10^−8^) (Table 3). Preoperative PSA, PSA density, cGS of 7 (3 + 4), 7 (4 + 3), and ≥8 were also significant factors for BCR-free survival (HR: 1.02 [95% CI: 1.01–1.03], 1.75 [1.40–2.19], 7.58 [1.47–39.1], 16.9 [3.96–72.1], and 28.8 [6.90–120.1], respectively), while age, BMI, and prostate volume were not. In multivariate analysis, the PCa-MRS score, preoperative PSA and cGS remained significant. Excluding cGS obtained from invasive procedures, PCa-MRS was the most significant factor for BCR. To evaluate the clinical usefulness of the PCa-MRS model, we categorized the patients into high and low score groups based on the probability score (Logit (P)) of the PCa-MRS model with a cutoff value of −0.82, which showed the most appropriate sensitivity (86.5%) and specificity (85.3%) (Supplementary Table 3) by Youden index. Patients with the high score showed significantly poorer BCR-free survival than those with the low score (*P* = 6.53 × 10^−10^) (Fig. 2a). High score group also showed significantly poorer overall survival than low score group (*P* = 1.53 × 10^−3^) (Fig. 2b).

**Table 3:** Results of Cox regression analysis of factors associated with BCR-free survival.

	Univariate		Multivariate^a^	
Variable	HR (95% CI)	*P*-value	HR (95% CI)	*P*-value
PCa-MRS				
High vs low score	4.23 (2.58–6.96)	1.25 × 10^−8^	2.26 (1.29–3.96)	0.004
Age	1.02 (0.98–1.06)	0.305		
BMI	1.05 (0.97–1.15)	0.246		
Prostate volume	1.00 (0.99–1.02)	0.736		
Preoperative PSA	1.02 (1.01–1.03)	2.49 × 10^−8^	1.01 (1.00–1.02)	0.014
PSA density	1.75 (1.40–2.19)	7.59 × 10^−7^		
Clinical Gleason score		4.43 × 10^−6^		1.74 × 10^−4^
7(3 + 4) vs ≤6	7.58 (1.47–39.1)	0.015	7.29 (1.41–37.7)	0.018
7(4 + 3) vs ≤6	16.9 (3.96–72.1)	1.34 × 10^−4^	12.6 (2.92–54.2)	0.001
≥8 vs ≤6	28.8 (6.90–120.1)	3.99 × 10^−6^	20.4 (4.80–86.4)	4.33 × 10^−5^

^a^Multivariate analysis was performed by Cox regression analysis with backward selection using PCa-MRS (categorical), preoperative PSA, and clinical Gleason score (categorical). To avoid the collinearity effect, PSA density was excluded from the multivariate analysis.

## Discussion

Predicting the risk of PCa metastasis and recurrence before starting treatment can minimize the overtreatment of indolent cases and help physicians choose appropriate treatment for high-risk ones. Circulating miRNAs from cell-free body fluids such as serum and urine have been demonstrated as noninvasive diagnostic or prognostic biomarkers for various cancers^7–9^. However, more robust and valid biomarkers are required for clinical application. This study had three aims: first, to discover urinary exosomal miRNAs associated with PCa metastasis; second, to develop a noninvasive, risk-scoring model for metastasis by using urinary exosomal miRNAs and clinical factors; and third, to validate the clinical applicability of this model.

We used urinary exosomes because urine can be collected easily and miRNAs are known to be enriched in urine-derived exosomes more than in cell-free urine^4,10^. In the discovery phase, we found six miRNAs differentially expressed between localized and metastatic PCas. Among them, three patterns (upregulation of miR-21, upregulation of miR-451, and downregulation of miR-636) were associated with metastasis. These associations were replicated in the validation phase and remained significant in the multivariate analysis, supporting the reliability of the associations.

MiR-21 is one of the commonly upregulated miRNAs in various human cancers and has been suggested as a universal biomarker^11^. It has also been recognized as an onco-miRNA that negatively regulated tumor-suppressor genes^12^. Elevated miR-21 was associated with BCR and metastatic hormone-refractory PCa^12,13^ and seemed to promote cancer progression and metastasis by enhancing TGF-β signaling^14^. Elevated miR-21 was found in serum of metastatic hormone-refractory PCa patients even with low levels of PSA^7^. However, in other studies, this pattern was not consistently reported^9,15^. In our study, the association of upregulated exosomal miR-21 with metastasis was consistently observed in discovery and validation sets. It shows the robustness of the association, but may be also partly because that miRNAs, particularly miR-21, are significantly enriched in urine-derived exosomes compared with cell-free urine^4,10,16^. To our knowledge, this is the first report on miR-21 overexpression in the urine of metastatic PCa patients.

MiR-451 has been reported to be dysregulated in diverse human cancers and to be involved in tumorigenesis^17^. However, its role in tumorigenesis is controversial. Moltzahn et al. found that serum miR-451 was elevated in the metastatic PCa patients^8^, which is consistent with our finding. In Watahiki et al.’s study comparing the miRNA expressions in the xenograft of metastatic and nonmetastatic PCa lines, miR-451 was upregulated in the metastatic line^18^. Panigrahi et al. reported that miR-451 was upregulated in PCa under the hypoxic condition, which is related to PCa progression and treatment failure^19^. These results strongly suggest that upregulated miR-451 would be involved in progression to PCa with poor prognosis. However, opposite findings were also reported. In some solid tumors, downregulated miR-451 is identified and suggested to have tumor-suppressor roles^20^. Further studies with larger samples will be required to clarify the roles of miR-451 in PCa.

MiR-636 expression in PCa has not been well studied. In one meta-analysis, miR-636 expression was downregulated in the recurrent PCa group after RP compared with the nonrecurrent group^21^, which is consistent with our results.

For reliable and clinically useful prediction of prognosis, we designed a risk-scoring model for PCa metastasis using the significant factors in the multivariate analysis (three miRNAs and preoperative PSA). The PCa-MRS model showed superior discriminatory power and higher sensitivity, specificity, and accuracy. The PCa-MRS model was also found to be more useful to predict BCR-free survival in the PCa patients who underwent RP than conventional clinical factors. Patients with higher PCa-MRS scores showed significantly poorer BCR-free survival than those with lower scores. To our knowledge, it is unique to combine miRNAs and clinical factors to develop a minimally invasive prediction model for PCa prognosis.

There are several limitations in our study. First, due to the limited sample size, there is a possibility of false-negative results, i.e., missing significant miRNA markers. To minimize the possibility of missing markers, we selected candidate miRNAs with less stringent criteria without multiple testing in the discovery phase. Second, for model building and survival analysis, we used the combined set of samples also due to the limited sample size. Although we performed an external validation of the PCa-MRS model using additional 37 PCa cases, further larger external validation will add more validity to our conclusion. Third, due to the heterogeneity of the exosomes from complex biological samples, some urinary exosomes might have been missed or over-represented. Fourth, we could not examine the urinary exosomal miRNAs after treatment due to lack of post-treatment sample.

In conclusion, our results showed the potential of urinary exosomal miRNAs as noninvasive markers for predicting metastasis and prognosis in PCa patients. Our risk-scoring model showed superior prediction performance to other models based on traditional markers such as PSA and can be more easily applied in clinical settings for stratifying patients’ risk of metastasis because it is minimally invasive.

## Methods

### Study subjects

Urine samples of PCa patients were obtained from the Korea Prostate Bank (2013.09–2020.08). Korea Prostate Bank is the nationally designated biobank that has been regularly inspected by the Ministry of Health and Welfare of Korea. They organized the “Standard Operating Procedures for the Korean Prostate Bank” considering the best practices for repositories released by the International Society for Biological and Environmental Repositories^22^. The biomaterials of included cases consisted of peripheral blood samples (plasma, serum, and buffy coat), urine samples, and tumor and normal tissues (frozen tissues, optimal cutting temperature [OCT]-embedded tissues, and paraffin-embedded tissues). Patient plasma, serum, buffy coat, and urine were obtained just before operation (surgical cases) or after a meeting with the physician (nonsurgical cases) after informed consent. We excluded patients who received neoadjuvant androgen deprivation therapy or had a prior history of another malignancy. Forty-two samples from 19 localized and 23 metastatic PCa patients, 70 samples from 56 localized and 14 metastatic PCa patients, and 37 samples from 27 localized and 10 metastatic PCa patients were used as discovery, independent validation of candidate miRNAs, and external model validation sets, respectively. We used a combined dataset from discovery and validation sets for the model construction, and a dataset combining all three sets for the survival analysis. This study was approved by the institutional review board of the Catholic University of Korea, College of Medicine (MC19SESI0020).

### Exosome preparation and RNA extraction

Exosomes were isolated from 5 mL of urine using the ExoQuick-TC exosome precipitation solution (System biosciences, Mountain View, CA) according to the manufacturer’s instruction. Briefly, urine was centrifuged at 3000 × *g* for 15 min to remove cells and debris. After collecting the supernatant, the exosome precipitation solution was added. The mixture was then incubated overnight at 4 °C and centrifuged at 1500 × *g* for 30 min at 4 °C. We aspirated the supernatant and spun down the residual solution via centrifugation at 1500 × *g* for 5 min to remove all traces of fluid. Total RNA was isolated from the pellets using TRIzol (Thermo Fisher Scientific, Waltham, MA), as described elsewhere^23^. Exosome markers CD63 (1:1000, Abcam, #ab134045) and CD9 (1:1000, Abcam, #ab92726) were checked in urine exosomes using western blot (Supplementary Fig. 4). Blot images were derived from the same experiment, respectively, and were processed in parallel.

### TLDA experiments and data analysis

For the discovery phase, miRNA profiles were examined using the TLDA (Thermo Fisher Scientific) according to the manufacturer’s instruction. Megaplex reverse transcription reactions and pre-amplification reactions were performed to increase the quantity of cDNA for miRNA expression analysis using the Megaplex PreAmp Primers Human Pool A and TaqMan PreAmp Master Mix (Thermo Fisher Scientific). MiRNA expression was evaluated via the TLDA panel A v2.0 (Thermo Fisher Scientific). Raw data were processed using the QuantStudio Real-Time PCR Software (Thermo Fisher Scientific) to determine a cycle threshold (Ct) value for each miRNA.

For TLDA data analysis, we selected U6 snRNA, one of the most stably expressed exosomal miRNAs in human urine^24^, as an internal control for normalization. The resultant ΔCt values were quantile normalized across all TLDA data using the HTqPCR R package from Bioconductor^25^. We excluded miRNAs that were unreliably quantified or expressed <30% in the discovery set from further analysis. MiRNAs with fold changes (metastatic/localized PCa) ≥±2 were considered differentially expressed between the two groups.

### miRNA-specific quantitative reverse transcription PCR

Expression of the candidate miRNAs chosen in the discovery phase was validated using the TaqMan microRNA Assay (miR-636-002088, miR-140-3p-002234, miR-21-5p-000397, miR-16-5p-000391, miR-142-3p-000464, and miR-451a-001141). U6 snRNA (U6 snRNA-001973) was used as an endogenous control. In brief, 5 µl of RNA was converted to first-strand cDNA with miRNA-specific primers using the TaqMan MicroRNA Reverse Transcription Kit (Thermo Fisher Scientific), followed by real-time PCR with the TaqMan probes. Relative expression of individual miRNAs in each case was defined as 2^−ΔCt^. We finally selected miRNAs that were expressed consistently in both TLDA and qRT-PCR. To validate the associations of miRNAs with metastasis, qRT-PCR was performed in the independent validation set using the TaqMan microRNA Assay. All qRT-PCR reactions were carried out in triplicate and samples with Ct>35 of U6 snRNA were excluded.

### Statistical analysis

Differences in the levels of the miRNAs in urinary exosomes by metastasis were compared using the Mann–Whitney test. The ROC curve and AUC were used to assess the predictive values of miRNA markers for metastasis. Based on the Youden index^26^ from the ROC curve, optimal cutoff values were calculated to compute sensitivity and specificity for each model (Youden’s *J* = sensitivity + specificity − 1). Univariate logistic regression was used to determine the association of clinical parameters and the miRNA levels with metastasis. Factors significantly associated with metastasis in the univariate analysis were entered into multivariate logistic regression analysis. We carried out 100 rounds of the five-fold cross-validation procedure to build and optimize the miRNA-based logistic regression predictive model named PCa-MRS. ROC analysis was performed to evaluate the predictive performance of the model comprising metastasis–related miRNA signatures. Probability was calculated for inclusion in the ROC analysis using the following formula: X = logit(P) = ln(P/1-P) = b_0_ + b_1_ΔCt1 + b_2_ΔCt2 + b_3_ΔCt3 + … + b_n_ΔCt_n_, where the b_i_ terms were the ith regression coefficients by logistic regression, and the ΔCt_i_ terms were the normalized Ct of each miRNA or preoperative PSA. The Delong method was used to compare the AUCs. Cox proportional hazards model was used to assess independent predictors of BCR-free survival using the PCa-MRS model and clinical variables. BCR-free survival was measured from the date of RP to relapse, which was defined as postoperative PSA level ≥ 0.2 ng/mL (a lower detection limit of 0.004 ng/mL) and successive PSA level ≥ 0.2 ng/mL after surgery, or to the date of last follow-up. Follow-up time was defined from the date of surgery with time of BCR as endpoints. Survival analysis for BCR was performed using the Kaplan–Meier method, and differences between the groups were analyzed using log-rank test. All statistical analyses were performed using glmnet R package^27^ and SPSS (version 21, Chicago, IL). All tests were two‐tailed, and noncorrected *P* < 0.05 was considered statistically significant.

### Reporting summary

Further information on research design is available in the Nature Research Reporting Summary linked to this article.

## Supplementary information

Supplementary Information

Reporting Summary

## Data Availability

High-throughput TLDA data have been deposited in NCBI’s Gene Expression Omnibus with the series accession number GSE173094 (https://www.ncbi.nlm.nih.gov/geo/query/acc.cgi?acc=GSE173094). Normalized Ct of each miRNA or preoperative PSA composing the model are available in Supplementary Table 4.

## References

[1] Stamey TA (2004). The prostate specific antigen era in the United States is over for prostate cancer: what happened in the last 20 years?. J. Urol..

[2] Sathianathen NJ, Konety BR, Crook J, Saad F, Lawrentschuk N (2018). Landmarks in prostate cancer. Nat. Rev. Urol..

[3] Urabe F, Kosaka N, Kimura T, Egawa S, Ochiya T (2018). Extracellular vesicles: toward a clinical application in urological cancer treatment. Int. J. Urol..

[4] Bhome R (2018). Exosomal microRNAs (exomiRs): small molecules with a big role in cancer. Cancer Lett..

[5] Nilsson J (2009). Prostate cancer-derived urine exosomes: a novel approach to biomarkers for prostate cancer. Br. J. Cancer.

[6] Rodriguez M (2017). Identification of non-invasive miRNAs biomarkers for prostate cancer by deep sequencing analysis of urinary exosomes. Mol. Cancer.

[7] Zhang HL (2011). Serum miRNA-21: elevated levels in patients with metastatic hormone-refractory prostate cancer and potential predictive factor for the efficacy of docetaxel-based chemotherapy. Prostate.

[8] Moltzahn F (2011). Microfluidic-based multiplex qRT-PCR identifies diagnostic and prognostic microRNA signatures in the sera of prostate cancer patients. Cancer Res..

[9] Stuopelyte K, Daniunaite K, Jankevicius F, Jarmalaite S (2016). Detection of miRNAs in urine of prostate cancer patients. Medicina (Kaunas.).

[10] Cheng L, Sun X, Scicluna BJ, Coleman BM, Hill AF (2014). Characterization and deep sequencing analysis of exosomal and non-exosomal miRNA in human urine. Kidney Int.

[11] Shi J (2016). Considering exosomal miR-21 as a biomarker for cancer. J. Clin. Med..

[12] Ribas J (2009). miR-21: an androgen receptor-regulated microRNA that promotes hormone-dependent and hormone-independent prostate cancer growth. Cancer Res..

[13] Li T (2012). miR-21 as an independent biochemical recurrence predictor and potential therapeutic target for prostate cancer. J. Urol..

[14] Bonci D (2016). A microRNA code for prostate cancer metastasis. Oncogene.

[15] Sapre N (2014). Curated microRNAs in urine and blood fail to validate as predictive biomarkers for high-risk prostate cancer. PLoS ONE.

[16] Fabris L, Calin GA (2016). Circulating free xeno-microRNAs—-the new kids on the block. Mol. Oncol..

[17] Pan X, Wang R, Wang ZX (2013). The potential role of miR-451 in cancer diagnosis, prognosis, and therapy. Mol. Cancer Ther..

[18] Watahiki A (2011). MicroRNAs associated with metastatic prostate cancer. PLoS ONE.

[19] Panigrahi GK (2018). Exosomal microRNA profiling to identify hypoxia-related biomarkers in prostate cancer. Oncotarget.

[20] Wang R (2011). MicroRNA-451 functions as a tumor suppressor in human non-small cell lung cancer by targeting ras-related protein 14 (RAB14). Oncogene.

[21] Pashaei E, Pashaei E, Ahmady M, Ozen M, Aydin N (2017). Meta-analysis of miRNA expression profiles for prostate cancer recurrence following radical prostatectomy. PLoS ONE.

[22] Campbell LD (2018). The 2018 Revision of the ISBER Best Practices: Summary of Changes and the Editorial Team’s Development Process. Biopreserv. Biobank.

[23] Tataruch-Weinert D, Musante L, Kretz O, Holthofer H (2016). Urinary extracellular vesicles for RNA extraction: optimization of a protocol devoid of prokaryote contamination. J. Extracell. Vesicles.

[24] Kinoshita T, Yip KW, Spence T, Liu FF (2017). MicroRNAs in extracellular vesicles: potential cancer biomarkers. J. Hum. Genet..

[25] Dvinge H, Bertone P (2009). HTqPCR: high-throughput analysis and visualization of quantitative real-time PCR data in R. Bioinformatics.

[26] Youden WJ (1950). Index for rating diagnostic tests. Cancer.

[27] Friedman J, Hastie T, Tibshirani R (2010). Regularization paths for generalized linear models via coordinate descent. J. Stat. Softw..

