# Supplementary material for: Urinary exosome microRNA signatures as a noninvasive prognostic biomarker for prostate cancer
Source: NPJ Genom Med. 2021 Jun 11;6:45. doi: 10.1038/s41525-021-00212-w (PMC8196022; doi:10.1038/s41525-021-00212-w)
Supplement: Supplementary file 1 — Supplementary Information [file 41525_2021_212_MOESM1_ESM.pdf]

1. Supplementary Figures

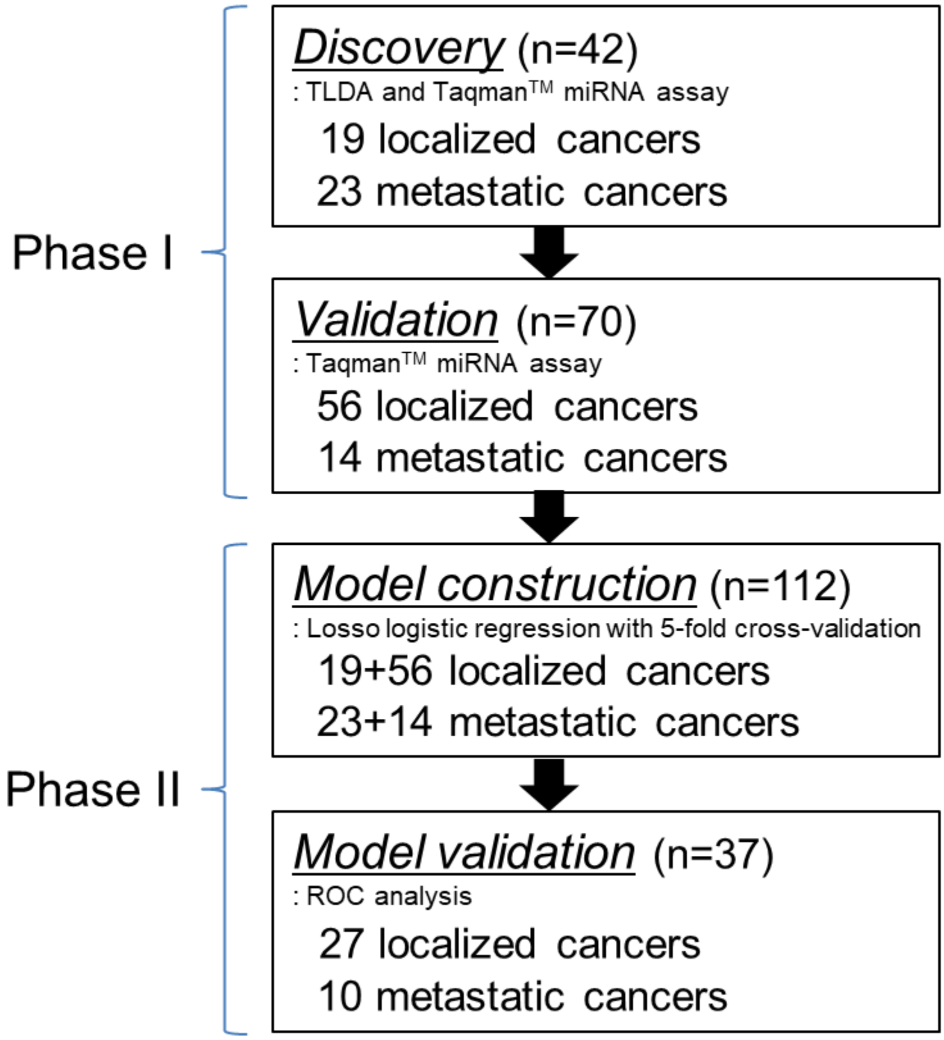

Supplementary Fig. 1. Overview of the study design for identifying miRNA markers of metastasis in prostate cancer. To identify miRNA markers as a signature for metastatic prostate cancer, this study was conducted in two phases; (I) discovery and independent validation of differentially expressed miRNAs by TaqMan low-density miRNA array (TLDA) and quantitative reverse transcription PCR (qRT-PCR) experiment; (II) model construction using Lasso logistic regression with 5-fold cross-validation and the external validation of the model. Statistical analysis was performed including risk score analysis and logistic regression model with ROC curves to evaluate the predictive capability of the candidate miRNAs towards metastasis with the combined set. ROC, receiver operating characteristic

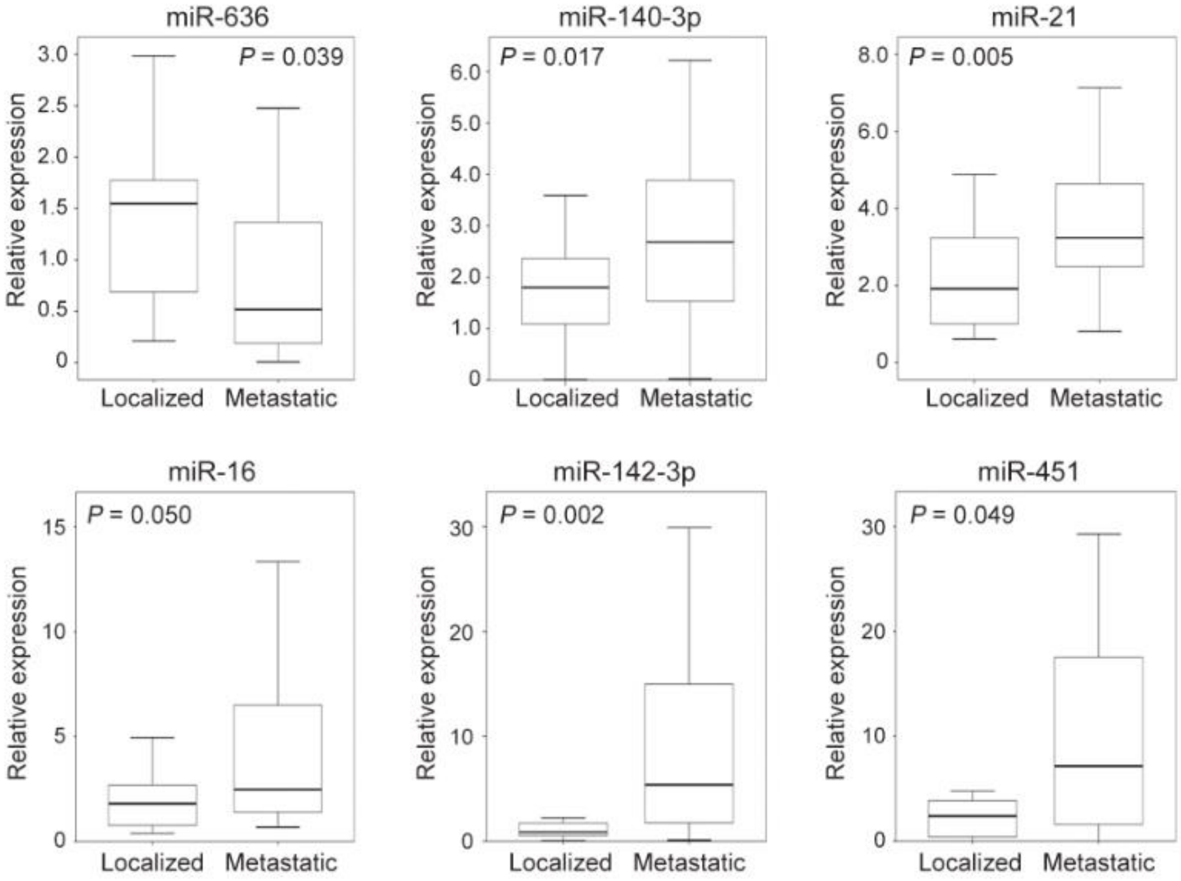

Supplementary Fig. 2. Relative expression of differentially expressed miRNAs in the discovery phase by Taqman low-density miRNA array. Data are presented as box plots of the median (and range) relative expression normalized to U6 snRNA.

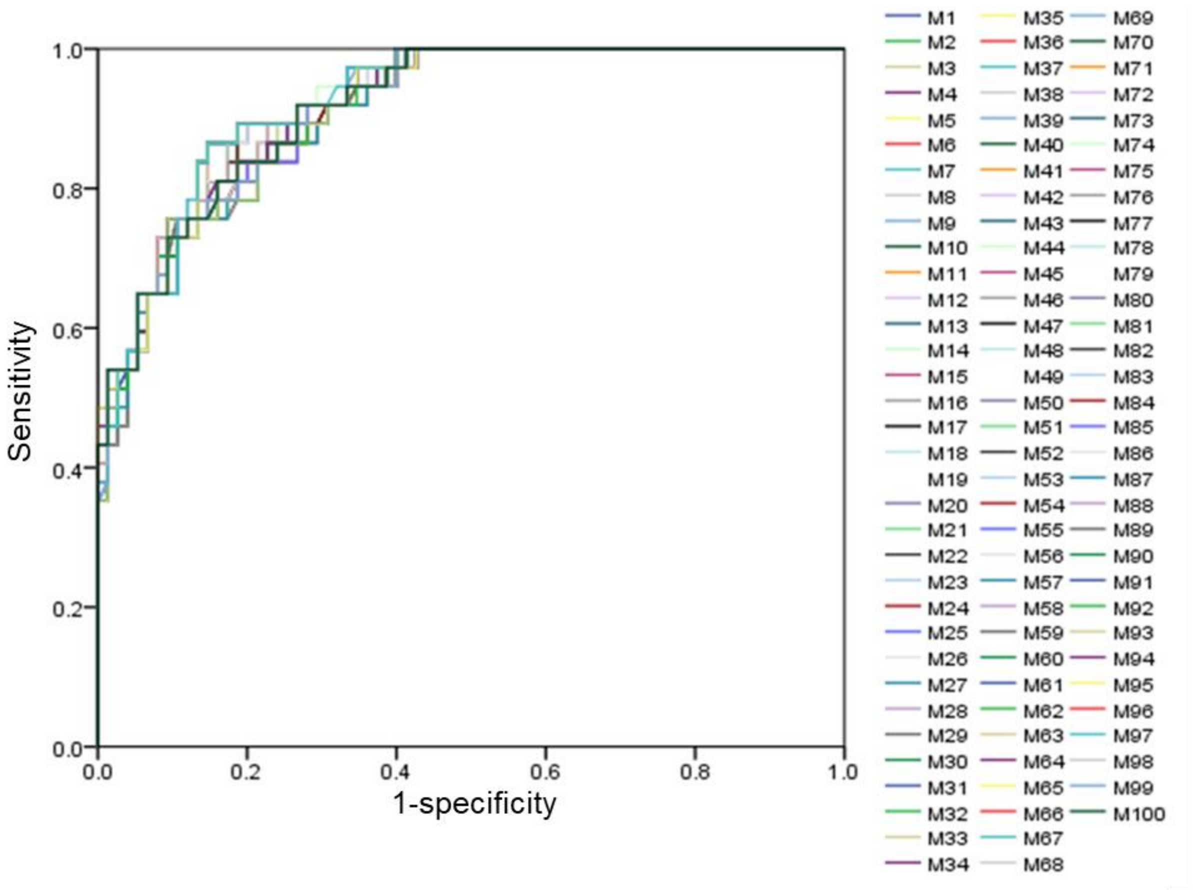

Supplementary Fig. 3. Performance of the 100 risk prediction models. The median AUC from 100 iterations of 5-fold cross-validation was 0.917 (range 0.909-0.925).

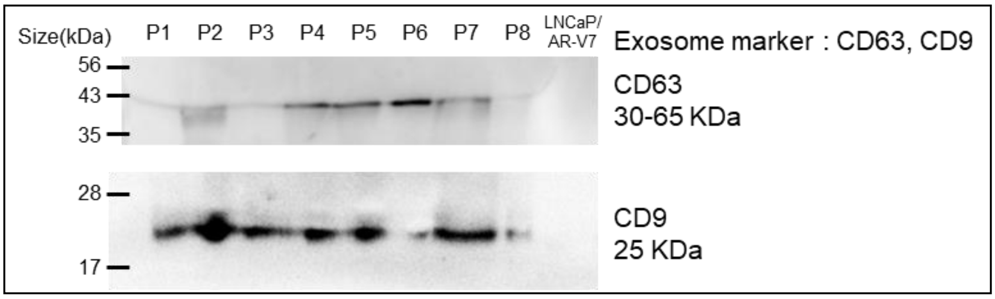

Raw blot images

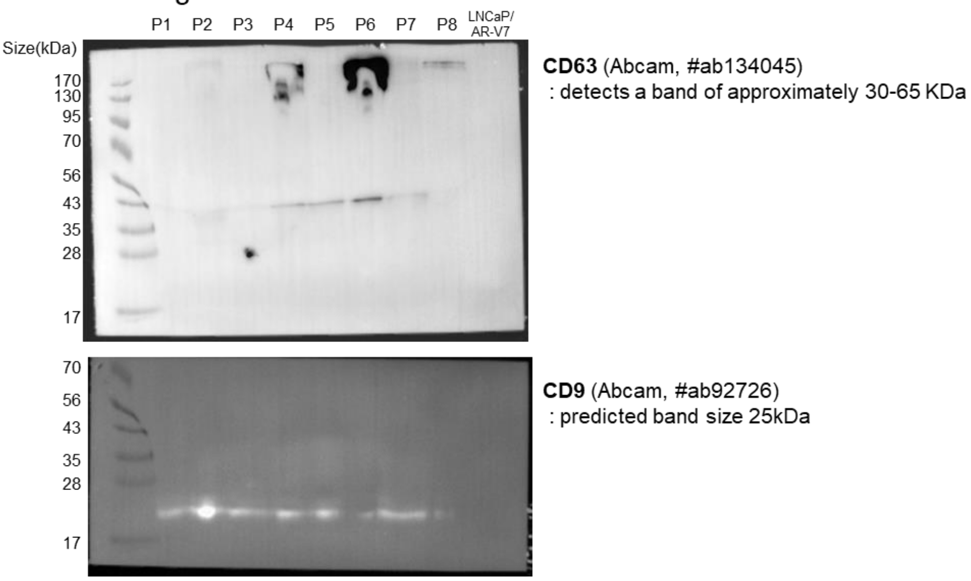

Supplementary Fig. 4. Verification of urine exosome. Examples of exosome markers (CD63 and CD9) were identified in urine exosomes using western blot. Blot images were derived from the same experiment respectively and were processed in parallel.

2. Supplementary Tables

Supplementary Table 1. Clinical characteristics of the study subjects

	**Discovery set**			**Validation set**			**External model validation set**		
	**Localized**	**Metastatic**	***P*-value**	**Localized**	**Metastatic**	***P*-value**	**Localized**	**Metastatic**	***P*-value**
Number	19	23		56	14		27	10	
Age, years*	74 (58-81)	74 (63-87)	0.593	71 (48-83)	71.5 (61-85)	0.825	68 (62-80)	63.5 (52-78)	0.054
BMI, kg/m2*	23.4 (17.6-29.4)	24.1 (16.5-31.1)	0.331	24.6 (18.0-30.9)	25.0 (20.6-28.7)	0.376	24.2 (20.8-30.1)	24.1 (17.5-29.2)	0.692
Preoperative PSA, ng/mL*	6.03 (2.52-11.3)	34.8 (4.60-1225)	$6.79x10^{-6}$	7.35 (0.17-152)	40.6 (4.04-701)	$9.20x10^{-5}$	7.37 (3.79-36.3)	22.6 (11.9-108)	$6.29x10^{-5}$
Prostate volume, mL*	35.6 (15.0-59.5)	36.5 (20.0-93.6)	0.627	31.6 (15.0-102)	35.0 (22.1-79.1)	0.384	42.5 (23.0-76.0)	49.4 (28.0-62.9)	0.311
PSA density, ng/mL^2	0.177 (0.076-0.69)	0.821 (0.082-16.5)	$9.40x10^{-5}$	0.247 (0.003-3.66)	1.44 (0.113-31.8)	0.017	0.212 (0.072-0.88)	0.925 (0.236-3.57)	0.001
Clinical Gleason score**			$5.04x10^{-10}$			0.008			0.024
≤6	18	1		15	0		9	0	
7 (3+4)	1	4		8	0		3	1	
7 (4+3)	0	7		15	3		4	6	
≥8	0	11		18	11		11	3	

* the values are represented as median with range.
** Analyzed by Fischer's exact test
BMI, body mass index; PSA, prostate-specific antigen.

Supplementary Table 2. List of 16 differentially expressed miRNAs between localized and metastatic prostate cancer patients in discovery set

Down-regulated miRNAs			Up-regulated miRNAs		
miRNA	Fold change	*P*-value	miRNA	Fold change	*P*-value
**hsa-miR-636-002088**	0.325	0.039	**hsa-miR-142-3p-000464**	4.326	0.002
hsa-miR-483-5p-002338	0.348	0.088	**mmu-miR-451-001141**	3.878	0.049
hsa-miR-330-000544	0.362	0.100	**hsa-miR-21-000397**	3.147	0.005
hsa-miR-29b-000413	0.419	0.929	**hsa-miR-16-000391**	2.951	0.050
hsa-miR-18a-002422	0.421	0.850	hsa-miR-126-002228	2.439	0.479
hsa-miR-363-001271	0.489	0.090	mmu-miR-491-001630	2.363	0.830
			hsa-miR-223-002295	2.292	0.093
			hsa-miR-210-000512	2.169	0.206
			**hsa-miR-140-3p-002234**	2.166	0.017
			hsa-miR-212-000515	2.044	0.129

Bold: $P<0.05$

Supplementary Table 3. Specificity, accuracy and threshold at specified sensitivity by model.

Sensitivity, %	Specificity, %				Accuracy, %				Threshold*			
	PCa-MRS	miRNA only	PSA only	cGS only	PCa-MRS	miRNA only	PSA only	cGS only	PCa-MRS	miRNA only	PSA only	cGS only
100	58.7	5.3	12.0	0.0	72.3	36.6	41.1	33.0	-2.19	-5.09	4.03	5
97.3	66.7	50.7	21.3	42.7	76.8	66.1	46.4	60.7	-1.71	-2.41	4.47	6
94.6	70.7	56.0	36.0		78.6	68.8	55.4		-1.66	-1.79	5.34	
91.9	73.3	72.0	45.3		79.5	78.6	60.7		-1.54	-1.08	6.22	
89.2	81.3	72.0	57.3		83.9	77.7	67.9		-1.09	-1.05	7.43	
86.5	85.3	76.0	57.3	56.0	85.7	79.5	67.0	66.1	**-0.82**	-0.86	7.47	**7 (3+4)**
83.8	86.7	76.0	60.0		85.7	78.6	67.9		-0.67	-0.78	7.66	
78.4	88.0	90.7	61.3		84.8	86.6	67.0		-0.38	**-0.23**	8.23	
75.7	89.3	90.7	66.7		84.8	85.7	69.6		-0.28	-0.20	9.25	
73.0	89.3	90.7	90.7		83.9	84.8	84.8		-0.13	-0.17	**14.0**	
64.9	94.7	92.0	92.0		84.8	83.0	83.0		0.53	0.14	22.2	
45.9	98.7	96.0	96.0		81.3	79.5	79.5		1.49	0.78	40.6	

*The thresholds with maximum Youden's *J* were indicated in bold for each model. (Youden's *J* = sensitivity + specificity -1)

PCa-MRS is the model with the highest AUC from the 100 iterations of Lasso logistic regression with 5-fold cross validation.
miRNA only model was derived using logistic regression with all 5 candidate miRNAs.
All models were constructed using the model construction set (75 localized and 37 metastatic PCa patients from the discovery and validation sets)
PCa-MRS model: -8.72 + (0.97 x ΔCt of miR-636) + (-0.49 x ΔCt of miR-21) + (-0.38 x ΔCt of miR-451) + (0.030 x preoperative PSA)
miRNA only model: -7.30 + (0.92 x ΔCt of miR-636) + (-0.67 x ΔCt of miR-21) + (-0.51 x ΔCt of miR-451) + (0.36 x ΔCt of miR-16) + (-0.16 x ΔCt of miR-142-3p)
PSA only model: preoperative prostate specific antigen only
cGS only model: clinical Gleason score only

Supplementary Table 4. Normalized expression values (delta Ct) of miR-636, miR-21 and miR-451 with preoperative PSA in this study

ID	Set	Feature	PSA	miR-636	miR-21	miR-451
UE1	discovery	localized	10.35	4.112	-8.245	0.106
UE2	discovery	localized	5.10	5.399	-2.204	6.650
UE3	discovery	localized	5.51	6.056	-2.493	5.582
UE4	discovery	localized	7.36	2.620	-3.905	2.622
UE5	discovery	localized	2.56	5.906	-4.837	1.686
UE6	discovery	localized	10.24	3.424	-4.266	4.059
UE7	discovery	localized	5.07	3.616	-5.300	-0.598
UE8	discovery	localized	6.11	5.733	-1.456	4.702
UE9	discovery	localized	9.16	4.916	-4.537	2.972
UE10	discovery	localized	8.23	7.174	-3.832	1.820
UE11	discovery	localized	6.03	3.573	-6.084	-2.328
UE12	discovery	localized	4.72	4.893	-2.667	-3.477
UE13	discovery	localized	4.03	3.852	-3.813	4.315
UE14	discovery	localized	7.42	3.934	-4.966	0.850
UE15	discovery	localized	5.04	4.804	-3.675	-1.09
UE16	discovery	localized	2.52	5.558	-2.421	8.602
UE17	discovery	localized	9.25	5.658	-6.414	-1.700
UE18	discovery	localized	3.21	4.966	-5.776	3.789
UE19	discovery	localized	11.31	4.943	-5.743	2.459
UE20	discovery	metastatic	4.60	3.981	-8.248	0.935
UE21	discovery	metastatic	64.31	4.859	-6.352	-2.620
UE22	discovery	metastatic	34.50	4.180	-6.917	0.691
UE23	discovery	metastatic	103.6	2.764	-8.462	-4.976
UE24	discovery	metastatic	7.47	5.562	-2.395	-1.152
UE25	discovery	metastatic	106	3.189	-4.396	-0.758
UE26	discovery	metastatic	702	4.809	-4.210	0.529
UE27	discovery	metastatic	178.8	6.402	-6.431	1.800
UE28	discovery	metastatic	144	4.330	-5.887	-1.447
UE29	discovery	metastatic	1225	5.047	-6.191	-4.250
UE30	discovery	metastatic	8.10	5.860	-3.594	2.918
UE31	discovery	metastatic	95.23	7.866	-4.851	2.174
UE32	discovery	metastatic	8.00	5.425	-5.850	3.523
UE33	discovery	metastatic	20.70	3.489	-6.970	-2.736
UE34	discovery	metastatic	5.40	4.554	-6.992	-4.211
UE35	discovery	metastatic	348	4.794	-7.039	-6.287
UE36	discovery	metastatic	15.42	5.623	-7.108	-1.189
UE37	discovery	metastatic	62.05	4.746	-4.894	-2.310
UE38	discovery	metastatic	8.40	4.342	-4.711	-3.338

UE39	discovery	metastatic	34.80	5.470	-6.395	0.147
UE40	discovery	metastatic	9.26	6.428	-6.970	1.763
UE41	discovery	metastatic	23.60	6.270	-6.894	-0.577
UE42	discovery	metastatic	78.60	6.908	-4.144	0.348
UE43	validation	localized	5.34	7.909	-3.962	4.551
UE44	validation	localized	4.14	5.118	-7.870	2.789
UE45	validation	localized	4.20	4.151	-5.840	3.093
UE46	validation	localized	3.93	5.327	-7.134	4.310
UE47	validation	localized	7.01	4.370	-5.532	4.529
UE48	validation	localized	7.63	5.594	-4.620	2.277
UE49	validation	localized	3.52	2.827	-3.829	1.003
UE50	validation	localized	11.93	5.398	-2.642	4.605
UE51	validation	localized	11.22	3.818	-3.948	1.158
UE52	validation	localized	9.63	4.323	-2.786	4.706
UE53	validation	localized	7.10	5.382	-2.386	2.931
UE54	validation	localized	4.47	4.356	-7.110	0.340
UE55	validation	localized	5.21	3.925	-5.662	-2.614
UE56	validation	localized	7.43	2.705	-2.913	7.888
UE57	validation	localized	4.35	3.502	-6.081	1.498
UE58	validation	localized	4.93	3.351	-3.993	2.406
UE59	validation	localized	7.66	2.923	-4.599	3.748
UE60	validation	localized	5.23	0.960	-7.661	-2.810
UE61	validation	localized	152.4	4.598	-4.934	1.926
UE62	validation	localized	37.00	6.183	-8.151	2.394
UE63	validation	localized	99.44	2.180	-7.191	-0.996
UE64	validation	localized	20.60	2.051	-7.867	1.472
UE65	validation	localized	27.50	3.091	-8.849	3.485
UE66	validation	localized	70.88	2.163	-4.458	3.288
UE67	validation	localized	32.32	1.395	-8.677	-0.574
UE68	validation	localized	7.00	5.973	-2.482	3.660
UE69	validation	localized	9.40	3.094	-3.964	3.069
UE70	validation	localized	7.39	2.173	-2.942	1.815
UE71	validation	localized	11.41	4.179	-5.937	1.352
UE72	validation	localized	0.17	2.291	-5.005	2.357
UE73	validation	localized	4.29	3.647	-6.374	0.568
UE74	validation	localized	10.45	4.174	-7.590	1.613
UE75	validation	localized	4.43	1.890	-7.231	1.008
UE76	validation	localized	7.30	2.744	-5.478	2.239
UE77	validation	localized	13.58	6.002	-2.705	-4.355
UE78	validation	localized	4.88	1.705	-4.396	1.131
UE79	validation	localized	14.02	4.567	-9.400	1.124

UE80	validation	localized	3.86	4.790	-7.104	-3.699
UE81	validation	localized	5.08	0.150	-8.101	3.116
UE82	validation	localized	9.86	4.772	-5.256	5.506
UE83	validation	localized	6.14	5.882	0.416	4.604
UE84	validation	localized	12.06	6.186	-2.152	8.924
UE85	validation	localized	3.23	6.225	-2.025	4.318
UE86	validation	localized	10.17	5.488	-3.867	3.162
UE87	validation	localized	5.50	4.687	-5.088	0.409
UE88	validation	localized	4.20	4.607	-5.517	2.246
UE89	validation	localized	4.66	5.086	-4.500	6.375
UE90	validation	localized	8.40	4.359	-3.262	5.141
UE91	validation	localized	13.58	8.790	-1.851	2.436
UE92	validation	localized	11.98	5.004	-3.731	1.646
UE93	validation	localized	6.22	5.657	-1.800	1.500
UE94	validation	localized	10.43	1.385	-9.096	-3.735
UE95	validation	localized	7.11	3.627	-5.803	-0.335
UE96	validation	localized	9.61	1.473	-8.450	3.977
UE97	validation	localized	5.59	4.491	-6.586	1.684
UE98	validation	localized	8.81	3.600	-9.605	2.520
UE99	validation	metastatic	41.60	4.663	-6.685	-0.894
UE100	validation	metastatic	29.85	4.308	-7.949	0.715
UE101	validation	metastatic	31.50	4.408	-7.442	0.405
UE102	validation	metastatic	7.00	6.215	-5.629	2.783
UE103	validation	metastatic	700.8	6.368	-4.872	-2.013
UE104	validation	metastatic	570.2	4.214	-5.226	-5.230
UE105	validation	metastatic	606.9	4.836	-1.870	4.659
UE106	validation	metastatic	7.57	7.329	-6.380	0.848
UE107	validation	metastatic	53.74	6.590	-5.468	5.945
UE108	validation	metastatic	15.00	5.521	-8.106	-3.352
UE109	validation	metastatic	47.28	4.315	-9.721	-0.451
UE110	validation	metastatic	39.60	3.353	-5.516	0.197
UE111	validation	metastatic	4.04	6.296	-8.187	-0.825
UE112	validation	metastatic	38.82	4.816	-9.291	0.424
UE113	model validation	localized	24.73	8.441	7.382	5.290
UE114	model validation	localized	13.70	-1.519	-13.018	-5.304
UE115	model validation	localized	6.18	9.212	1.807	4.115
UE116	model validation	localized	5.31	2.074	0.319	8.212
UE117	model validation	localized	8.25	5.407	-3.034	-1.905
UE118	model validation	localized	8.90	2.753	0.489	1.290
UE119	model validation	localized	5.87	5.438	-2.822	2.620
UE120	model validation	localized	4.41	5.469	-1.668	7.054

UE121	model validation	localized	6.74	4.853	-2.951	-0.097
UE122	model validation	localized	7.46	9.798	1.277	3.130
UE123	model validation	localized	6.80	8.500	2.286	9.585
UE124	model validation	localized	6.10	9.261	-0.600	4.801
UE125	model validation	localized	5.49	8.803	-2.251	3.660
UE126	model validation	localized	7.37	4.872	-2.573	-1.121
UE127	model validation	localized	4.81	2.938	-0.355	-1.268
UE128	model validation	localized	3.79	6.749	-4.763	3.266
UE129	model validation	localized	8.00	-0.982	-4.061	0.514
UE130	model validation	localized	13.89	4.768	-2.446	4.862
UE131	model validation	localized	12.36	1.892	-3.257	7.838
UE132	model validation	localized	19.00	3.541	2.550	11.503
UE133	model validation	localized	36.30	7.162	-3.467	-0.829
UE134	model validation	localized	8.15	5.643	2.712	11.007
UE135	model validation	localized	6.99	6.263	-3.623	6.065
UE136	model validation	localized	10.44	2.685	-4.091	-0.263
UE137	model validation	localized	26.29	6.276	-1.840	1.162
UE138	model validation	localized	5.31	6.441	-4.146	-0.816
UE139	model validation	localized	4.93	5.558	-2.036	2.960
UE140	model validation	metastatic	100	5.501	-6.439	-6.201
UE141	model validation	metastatic	21.30	6.995	-1.045	6.177
UE142	model validation	metastatic	51.89	5.060	-6.153	0.780
UE143	model validation	metastatic	17.08	8.255	-2.404	4.472
UE144	model validation	metastatic	100	3.638	-5.193	-4.152
UE145	model validation	metastatic	108.4	12.457	1.318	10.753
UE146	model validation	metastatic	15.10	6.582	-3.578	3.895
UE147	model validation	metastatic	13.00	7.918	-4.979	-0.461
UE148	model validation	metastatic	23.80	2.633	-6.636	-6.540
UE149	model validation	metastatic	11.90	4.236	-8.765	-4.963
