# Supplementary material for: Urinary exosome microRNA signatures as a noninvasive prognostic biomarker for prostate cancer
Source: NPJ Genom Med. 2021 Jun 11;6:45. doi: 10.1038/s41525-021-00212-w (PMC8196022; doi:10.1038/s41525-021-00212-w)
Supplement: Supplementary file 2 — Reporting Summary [file 41525_2021_212_MOESM2_ESM.pdf]

nature research

Corresponding author(s): Yeun-Jun Chung

Last updated by author(s): May 18, 2021

Reporting Summary

Nature Research wishes to improve the reproducibility of the work that we publish. This form provides structure for consistency and transparency in reporting. For further information on Nature Research policies, see our Editorial Policies and the Editorial Policy Checklist.

Statistics

For all statistical analyses, confirm that the following items are present in the figure legend, table legend, main text, or Methods section.

n/a	Confirmed	
☐	☒	The exact sample size (*n*) for each experimental group/condition, given as a discrete number and unit of measurement
☐	☒	A statement on whether measurements were taken from distinct samples or whether the same sample was measured repeatedly
☐	☒	The statistical test(s) used AND whether they are one- or two-sided<br>*Only common tests should be described solely by name; describe more complex techniques in the Methods section.*
☐	☒	A description of all covariates tested
☐	☒	A description of any assumptions or corrections, such as tests of normality and adjustment for multiple comparisons
☐	☒	A full description of the statistical parameters including central tendency (e.g. means) or other basic estimates (e.g. regression coefficient) AND variation (e.g. standard deviation) or associated estimates of uncertainty (e.g. confidence intervals)
☐	☒	For null hypothesis testing, the test statistic (e.g. *F*, *t*, *r*) with confidence intervals, effect sizes, degrees of freedom and *P* value noted<br>*Give P values as exact values whenever suitable.*
☒	☐	For Bayesian analysis, information on the choice of priors and Markov chain Monte Carlo settings
☐	☒	For hierarchical and complex designs, identification of the appropriate level for tests and full reporting of outcomes
☒	☐	Estimates of effect sizes (e.g. Cohen's *d*, Pearson's *r*), indicating how they were calculated

Our web collection on statistics for biologists contains articles on many of the points above.

Software and code

Policy information about availability of computer code

Data collection	Raw Ct data of TLDA and TaqMan microRNA assays were processed using QuantStudio Real-Time PCR Software (Thermo Fisher Scientific).
Data analysis	TLDA data analysis : Quantile-normalized ΔCt against U6 snRNA and fold-change between the localized and metastatic cancer across all TLDA data using the HTqPCR R package from Bioconductor.<br><br>Model construction with internal cross-validation : We conducted 100 iterations of Lasso logistic regression with 5-fold cross-validation to build and optimize the miRNA-based logistic regression predictive model using glmnet R package.<br><br>Statistical analysis : Other statistical analyses including Mann-Whitney test, ROC analysis and survival analysis were performed using SPSS (version 21).

For manuscripts utilizing custom algorithms or software that are central to the research but not yet described in published literature, software must be made available to editors and reviewers. We strongly encourage code deposition in a community repository (e.g. GitHub). See the Nature Research guidelines for submitting code & software for further information.

Data

Policy information about availability of data

All manuscripts must include a data availability statement. This statement should provide the following information, where applicable:

- Accession codes, unique identifiers, or web links for publicly available datasets
- A list of figures that have associated raw data
- A description of any restrictions on data availability

High-throughput TLDA data that is associated with Supplementary Figure 2 in this study can be found at NCBI's Gene Expression Omnibus (series accession number:

GSE173094; https://www.ncbi.nlm.nih.gov/geo/query/acc.cgi?acc=GSE173094). TaqMan miRNA assay data associated with Figures 1A/B and 2 can be found in Supplementary Table 3 and 4.

Field-specific reporting

Please select the one below that is the best fit for your research. If you are not sure, read the appropriate sections before making your selection.

☒ Life sciences ☐ Behavioural & social sciences ☐ Ecological, evolutionary & environmental sciences

For a reference copy of the document with all sections, see nature.com/documents/nr-reporting-summary-flat.pdf

Life sciences study design

All studies must disclose on these points even when the disclosure is negative.

Sample size	Urine samples from a total of 149 PCa patients were evaluated.<br>Forty-two samples from 19 localized and 23 metastatic PCa patients, 70 samples from 56 localized and 14 metastatic PCa patients, and 37 samples from 27 localized and 10 metastatic PCa patients were used as discovery, independent validation of candidate miRNAs, and external model validation sets, respectively.
Data exclusions	We excluded miRNAs that were unreliably quantified or expressed < 30% in the discovery set (TLDA data analysis) from further analysis.<br>Samples with Ct>35 of U6 snRNA were excluded.
Replication	All qRT-PCR reactions were carried out in triplicate.
Randomization	This study is a retrospective biomarker analysis using banked samples from patients diagnosed with localized or metastatic prostate cancer. We did not carry out a truly randomization, but we confirmed the distribution of clinical variables such as age and BMI for each group. Additionally, we conducted 100 rounds of the 5-fold cross-validation procedure during the model construction to provide more persuasive evidence. It should be considered exploratory and be interpreted with caution given the retrospective nature and the small number of samples.
Blinding	YHP, JYL and MYK participated in acquisition of data, sample preparation and clinical reviews. SS, SHJ and SHJ carried out the molecular genetic studies and following analysis. Samples collection and data analysis were conducted by separate investigators, and hence it was blinded.

Reporting for specific materials, systems and methods

We require information from authors about some types of materials, experimental systems and methods used in many studies. Here, indicate whether each material, system or method listed is relevant to your study. If you are not sure if a list item applies to your research, read the appropriate section before selecting a response.

Materials & experimental systems

n/a	Involved in the study	
☐	☒	Antibodies
☒	☐	Eukaryotic cell lines
☒	☐	Palaeontology and archaeology
☒	☐	Animals and other organisms
☐	☒	Human research participants
☒	☐	Clinical data
☒	☐	Dual use research of concern

Methods

n/a	Involved in the study	
☒	☐	ChIP-seq
☒	☐	Flow cytometry
☒	☐	MRI-based neuroimaging

Antibodies

Antibodies used	Anti-CD9 antibody [EPR2949] (ab92726, Abcam)<br>Anti-CD63 antibody [EPR5702] (ab134045, Abcam)
Validation	Anti-CD9 antibody [EPR2949] (ab92726, Abcam) : Rabbit monoclonal [EPR2949] to CD9. Produced recombinantly (animal-free) for high batch-to-batch consistency and long term security of supply. Reacts with: Mouse, Rat, Human<br>Anti-CD63 antibody [EPR5702] (ab134045, Abcam) : Rabbit monoclonal [EPR5702] to CD63. Produced recombinantly (animal-free) for high batch-to-batch consistency and long term security of supply. Reacts with: Human

Human research participants

Policy information about studies involving human research participants

Population characteristics	Human urine samples were obtained from 149 (male) prostate cancer (PCa) patients aged 48-83, with BMI ranging 17.6-31.1 kg/m2. 102 out of the 149 patients were diagnosed with localized PCa, and 47 patients were metatstatic. Among the 149 PCa patients, we examined 136 PCa patients who received radical prostatectomy for recurrence. The median duration of follow-up for BCR-free survival was 28.5 months (ranging 0.37-88.5 months).
Recruitment	Urine samples of PCa patients were obtained from the Korea Prostate Bank (2013.09-2020.08). It was obtained just before operation (surgical cases) or after a meeting with the physician (non-surgical cases) after informed consent. We excluded patients who received neoadjuvant androgen deprivation therapy or had a prior history of another malignancy to avoid bias in recruiting participants.
Ethics oversight	This study was approved by the institutional review board of the Catholic University of Korea, College of Medicine (MC19SESI0020).

Note that full information on the approval of the study protocol must also be provided in the manuscript.
